# The Effect of High-Intensity Interval Training on Exercise Capacity in Patients with Coronary Artery Disease: A Systematic Review and Meta-Analysis

**DOI:** 10.1155/2023/7630594

**Published:** 2023-04-03

**Authors:** Siyi Li, Xiankun Chen, Huachen Jiao, Yan Li, Guanghui Pan, Xue Yitao

**Affiliations:** ^1^The First Clinical Medical School, Shandong University of Traditional Chinese Medicine, Jinan, China; ^2^State Key Laboratory of Dampness Syndrome of Chinese Medicine, The Second Affiliated Hospital of Guangzhou University of Chinese Medicine, Guangzhou, China; ^3^Key Unit of Methodology in Clinical Research, Guangdong Provincial Hospital of Chinese Medicine, Guangzhou, China; ^4^Department of Global Public Health, Health Systems and Policy, Karolinska Institutet, Stockholm 17177, Sweden; ^5^Affiliated Hospital of Shandong University of Traditional Chinese Medicine, Jinan 250014, China

## Abstract

**Background:**

The optimal exercise prescription for coronary artery disease (CAD) remains under debate. The aim of our meta-analysis is to investigate the efficacy of high-intensity interval training (HIIT) versus moderate-intensity continuous training (MICT) of coronary artery disease patients.

**Methods:**

Electronic databases were searched from their inception date until October 23, 2021, and the articles include randomized controlled trials. The mean differences and 95% confidence intervals were calculated, and heterogeneity was assessed using the *I*^2^ test.

**Results:**

The study standards were met by seventeen studies. The pooled studies included 902 patients. HIIT resulted in improvement in peak oxygen uptake (1.50 ml/kg/min, 95% confidence interval: 0.48 to 2.53, *n* = 853 patients, and low quality evidence) compared with MICT. There was no discernible difference between the individuals in the HIIT group and the MICT group in terms of systolic/diastolic blood pressure or peak/resting heart rate.

**Conclusion:**

This systematic review and meta-analysis reported the superiority of HIIT versus MICT in enhancing peak oxygen uptake in CAD patients.

## 1. Introduction

The main cause of death worldwide has been coronary artery disease (CAD) [1]. Cardiac rehabilitation (CR) based on exercise training is an approach to enhance cardiopulmonary capacity, metabolic parameters, and quality of life [2]. CR in patients with CAD decreases angina [3], hospitalizations [4], and mortality [5].

According to the intensity and method of training protocols, interrelated exercise rehabilitation can be divided into high-intensity interval training (HIIT) and moderate-intensity continuous exercise (MICT). MICT has shown some advantages in decreasing the cardiovascular risk and mortality [6]. Due to the exercise protocol of MICT, there remains a low level of compliance with CR. In 2007, the American Heart Association recommended HIIT, which consists of repetition of quick and intense bursts of exercise, followed by short recovery periods [7].

In recent years, a growing amount of evidence proved that HIIT has beneficial effects on exercise capacity and cardiovascular function. However, these studies were limited by the small sample size and short follow-up period. Therefore, there is not sufficient clinical evidence to prove the efficiency of HIIT in CAD patients. Previous systematic reviews [8–10] also showed the superiority of HIIT on exercise capacity in patients involved with an exercise-based cardiac rehabilitation program. However, the most updated systematic review performed their literature search in November 2016 [11]. The study has since been followed by the publication of new studies.

The objective of this systematic review with meta-analysis was to evaluate the benefits of HIIT compared with MICT. In addition, we evaluated for the effects of HIIT on exercise capacity, blood pressure, and heart rate in CAD patients.

## 2. Methods

This systematic review was conducted and reported in accordance with the Preferred Reporting Items for Systematic Reviews and Meta-Analyses statement (Supplementary Materials: PRISMA 2009 Checklist) [12] and the Cochrane Handbook for Interventional Reviews [13]. The study protocol has been published previously in INPLASY, the registration number is INPLASY202240036 (available in https://inplasy.com/inplasy-2022-4-0036/).

### 2.1. Search Strategy

The electronic databases PubMed, Cochrane Central Register of Controlled Trials (CENTRAL), EMBASE, and CINAHL were searched from their inception until October 23, 2021. The searches were restricted to articles written in English. The search strategy details are provided in the Supplemental Materials—search strategy.

### 2.2. Study Selection

The full text was reviewed of all included articles. Two reviewers (S. L. and X. C.) independently screened the titles and abstracts. Furthermore, full-text screening was conducted according to the criteria for inclusion and exclusion. Disagreements for inclusion were discussed by the two reviewers and resolved by senior authors (Y. X.). Randomized controlled trials (RCTs) were included and the selection criteria are described below. The inclusion criteria were as follows: (1) RCTs comparing the effectiveness of HIIT with MICT in participants with CAD; (2) at least one of the following outcomes were measured—VO_2peak_, peak heart rate (HRpeak), resting heart rate (HRrest), resting systolic blood pressure (SBP), and resting diastolic blood pressure (DBP); and (3) the language was restricted to English. The exclusion criteria were as follows: (1) single-arm research and animal experiment research; (2) conference papers, letters, or abstracts where the full text was not available; and (3) incomplete data.

### 2.3. Data Collection

The data extraction form was predefined and included the following: population characteristics, intervention duration, training protocols, and outcome measures. One reviewer (S. L) used a standardized form to extract data from the included articles, and the extracted data were checked by a second reviewer (X. C). Attempts were made to contact the original investigators regarding any missing data. Any discrepancies were resolved by agreement after rechecking the source papers and via further discussion with a third reviewer (Y. X.).

### 2.4. Risk of Bias Assessment

In accordance with the recommendations in the Cochrane Handbook, the trials' methodological quality was independently evaluated by two reviewers (S. L. and X. C) using the Cochrane risk of bias assessment tool. Any discrepancies were resolved by agreement after rechecking the source papers and further discussion with a third reviewer (Y. X.). The following domains were considered: (1) random sequence generation, (2) allocation concealment, (3) blinding of the patients and personnel, (4) blinding of the outcome assessors for the primary outcomes, (5) incomplete outcome data, (6) selective reporting, and (7) other bias.

### 2.5. Quality of Evidence

The Grading of Recommendations, Assessment, Development, and Evaluation (GRADE) [14] was used to assess the quality of evidence of outcomes, which criteria comprised the risk of bias, inconsistency, indirectness, inaccuracy, and publication bias. The quality of evidence was classified as high, moderate, low, or very low.

### 2.6. Statistical Analysis

Statistical analysis was performed with Review Manager (RevMan, Version 5.4.1 The Cochrane Collaboration, Copenhagen, Denmark) [15]. Given that all of the variables in the included studies consisted of continuous data, we used the mean difference (MD) when the same instrument was used, or the standardized mean difference (SMD) when different instruments were used, with 95% confidence intervals (CI) to analyze the outcomes. A *p* value < 0.05 was considered statistically significant. Heterogeneity was assessed with a chi-squared test (*p* < 0.10 was considered indicative of statistical significance) and the *I*^2^ statistic (where *I*^2^ > 25%, 50%, and 75% indicated moderate, substantial, or considerable heterogeneity, respectively). When *I*^2^ is less than 50%, it indicated low heterogeneity, and a fixed-effects model would be chosen; otherwise, a random-effects model was adopted. Potential publication bias was evaluated by visual examination of funnel plot asymmetry and Egger's test (a *p* value < 0.05 was considered statistically significant). When the number of articles included in one analysis was limited (i.e., less than 10), the risk for publication bias was not assessed.

## 3. Result

### 3.1. Study Selection

**Figure 1:**
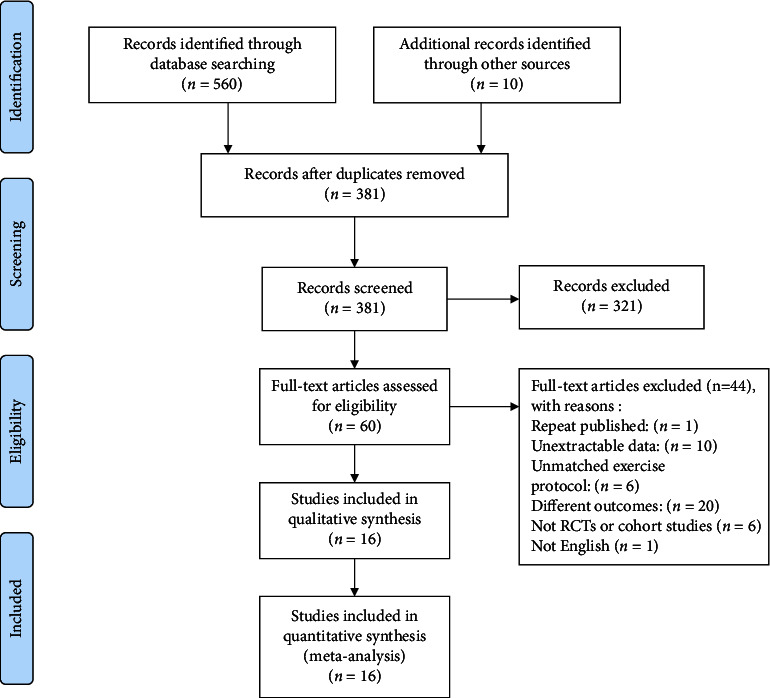
Flowchart of study identification and selection.

The process of study selection is shown in Figure 1. The initial search identified 570 articles (560 from the database search and 10 from the manual search), of which 381 were eligible for title and abstract scanning following the exclusion of duplicates. Based on the inclusion and exclusion criteria, 321 studies were excluded with 60 remaining. After the full texts of 60 articles were completely read, 16 articles met the eligibility criteria and were included in the meta-analysis [16–31].

### 3.2. Characteristics of Included Studies

Table 1 lists the general characteristics of the included studies, and the studies consisted of seven RCTs and one retrospective cohort study. One study [16] had a three-arm parallel group design. A total of sixteen studies comprising 853 patients were included for the analysis, and 406 patients underwent HIIT. The number of participants included in each study in our meta-analysis ranged from 14 to 174, and the mean age of the included participants ranged from 55.9 to 68 years. In the included studies, MICT was applied for the intervention of the control group. The duration of the interventions ranged between 4 and 12 weeks.

### 3.3. Risk of Bias

**Figure 2:**
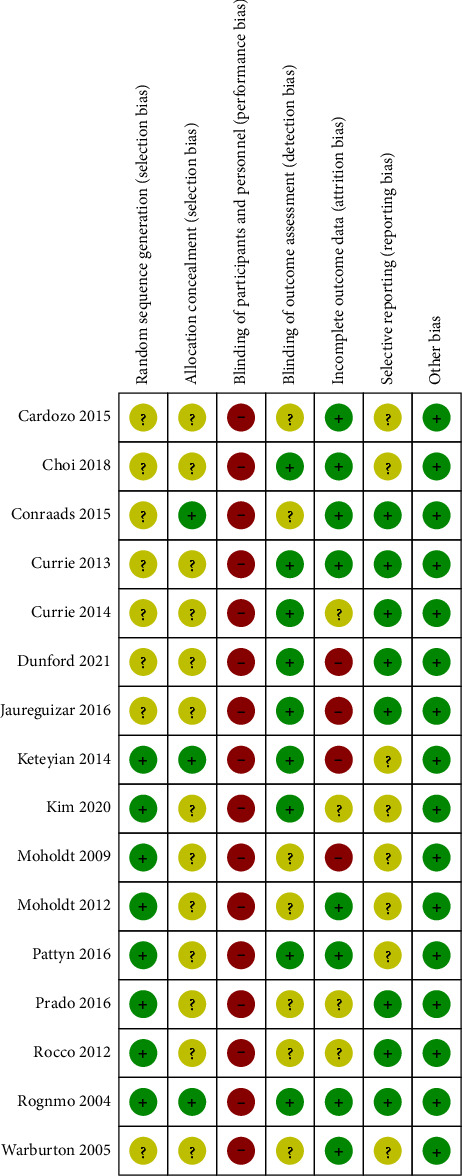
Risk of bias summary.

The individual items on the risk of bias assessment are shown in Figure 2. Sixty percent of the included RCTs provided adequate random sequence generation but only four studies reported allocation concealment methods. As both HIIT and MICT are exercise trainings, designing an experiment with a credible placebo-control arm is challenging. Thus, all RCTs were open label. All studies claimed that the outcome assessors had been blinded to the patient treatment allocation. Four studies [21–23, 26] reported incomplete outcome data because the participants were lost to follow-up, and the reasons for loss or withdrawal were noted in the literature. Approximately 50% of the included studies were at unclear risk of selective reporting because neither their protocol nor trial registration information was available.

The risk of publication bias, as analyzed by funnel plots, showed only minor asymmetry (Supplementary Figure S1). Thus, a publication bias mechanism is not a major cause of concern.

### 3.4. Quality of Evidence

The GRADE system showed that the quality of evidence was low for VO_2peak_ because of unclear allocation concealment or lack of blinding. The quality of evidence was downgraded to very low for the SBP, DBP, and heart rate because of the large heterogeneity and risk of bias.

### 3.5. Meta-Analysis of Outcomes

#### 3.5.1. Peak Oxygen Uptake

**Figure 3:**
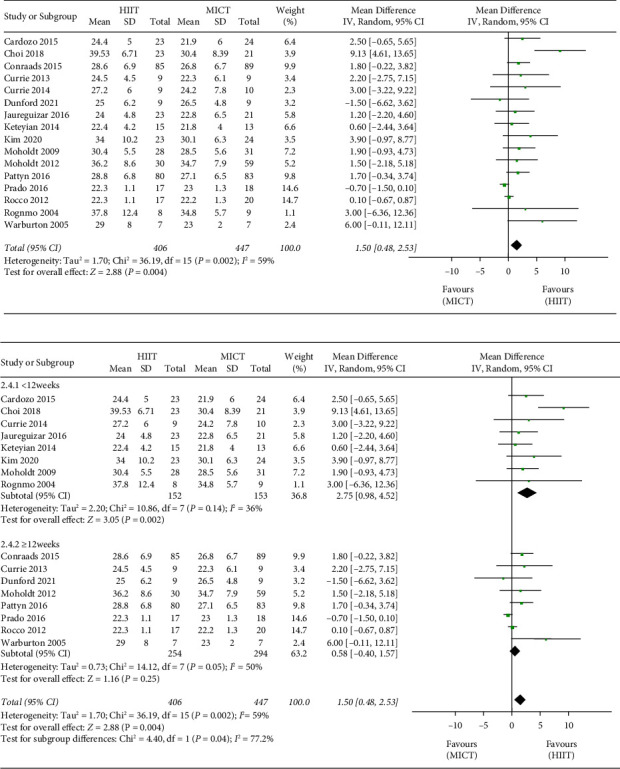
Meta-analysis results for VO_2peak_ (mL/kg/min).

VO_2peak_ was measured in 16 studies [16–31] with a total of 853 patients. The pooled results showed that HIIT led to a statistically significant 1.50 mL/kg/min improvement in the patients' VO_2peak_ (95% CI, 0.48 to 2.53; *I*^2^ = 59%; Figure 3(a)). A subgroup analysis was performed on the duration of intervention (<12 and ≥ 12 weeks) for HIIT versus MICT on VO_2peak_. The short-term group (<12 weeks) showed a significant improvement in VO_2peak_ (MD = 2.75 mL/kg/min, 95% CI, 0.98, 4.52; *I*^*2*^ = 36%; Figure 3(b)). The analysis long-term group (≥12 weeks) showed no significant effect on VO_2peak_ (MD = 0.58 mL/kg/min, 95% CI, −0.40, 1.57; *I*^2^ = 50%; Figure 3(b)).

#### 3.5.2. Blood Pressure

**Figure 4:**
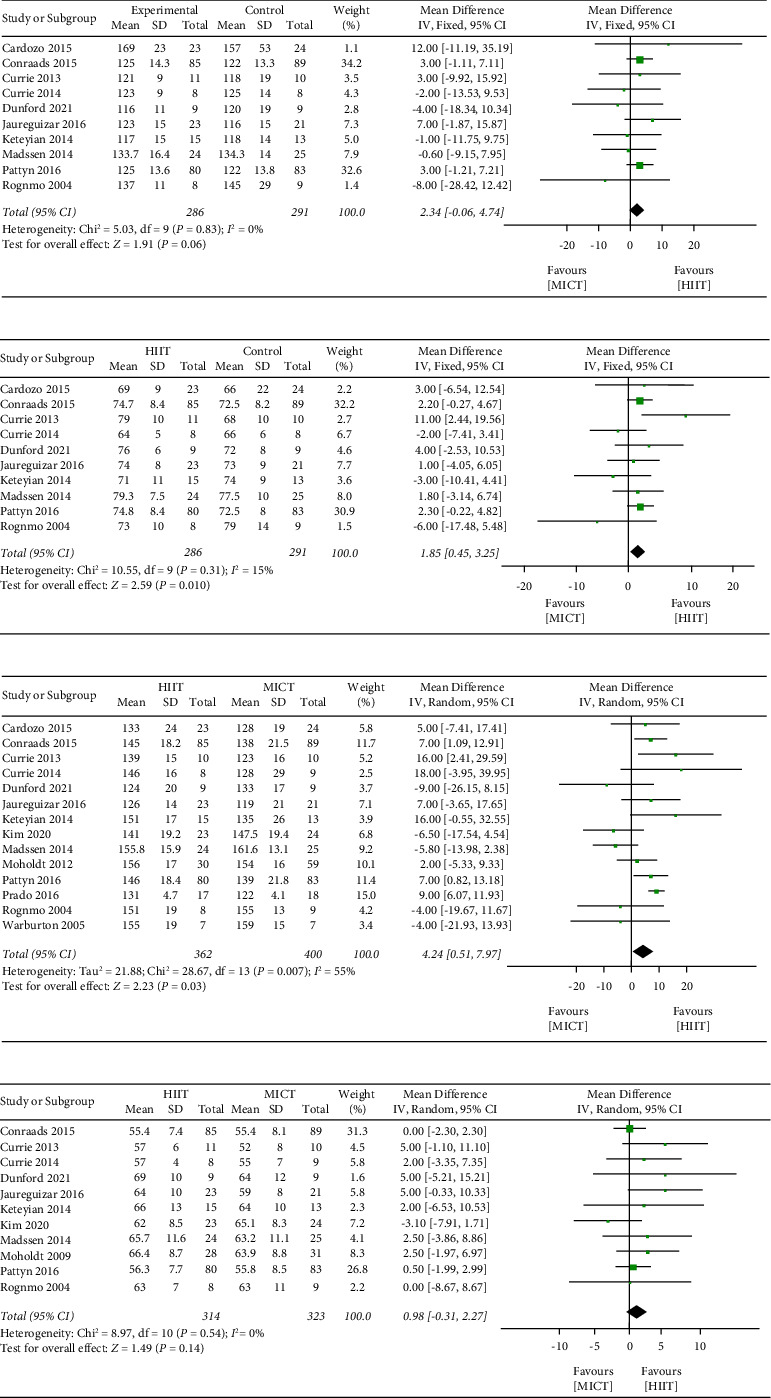
Meta-analysis results for (a) SBP (mmHg), (b) DBP (mmHg), (c) HR peak (mmHg), and (d) HR rest (mmHg).

Blood pressure included SBP and DBP, which were measured in 9 studies [16, 18–23, 27, 30]with a total of 528 patients. The results of our meta-analysis indicated a small but significant benefit from HIIT on SBP (MD = 2.59 mmHg; 95% CI, 0.09 to 5.09; *I*^2^ = 0%; Figure 4(a)). Moreover, the beneficial effect of HIIT on DBP was also small but significant (MD = 1.86 mmHg, 95% CI: 0.40 to 3.32; *I*^2^ = 24%; Figure 4(b)).

#### 3.5.3. Heart Rate

HRpeak was available for 13 [16, 18–25, 27, 28, 30, 31] studies with a total of 713 patients. The pooled results showed that HIIT led to a statistically significant increase in Hrpeak (MD = 5.51 bpm; 95% CI, 2.13 to 8.89), but the heterogeneity was considerable (*I*^2^ = 40%; Figure 4(c)). HRrest was available for 10 [18–22, 24, 26, 27, 30] studies with a total of 588 patients. The results of the meta-analysis indicated no significantly greater effect from HIIT on HRrest (MD = 0.19 bpm; 95% CI, −0.40 to 2.23; Figure 4(d)).

## 4. Discussion

The overall results of this study, which includes data from 16 RCTs and 853 patients, confirm a significantly larger effect size for VO_2peak_ (+1.50 ml/min/kg) in favor of HIIT. But the results of our meta-analysis found no significant effect on SBP and DBP, or HRpeak and HRrest. Although the meta-analysis of each outcome shows a certain degree of heterogeneity (*I*^2^<50%), we also used the random effect model, sensitivity analysis, and subgroup analysis to indicate the robustness of the results. Therefore, the results of our meta-analysis are relatively reliable.

Aerobic exercise has long been the cornerstone of cardiac rehabilitation programs for patients with CAD, and improving the aerobic exercise capacity of patients with CAD is its most significant benefit [31]. Aerobic exercise capacity is the strongest predictor of all cardiovascular morbidity and mortality and is the process of uptake, transport, and utilization of oxygen [5, 18]. In recent decades, MICT has been recommended for CAD patients according to the guidelines [32]. Several studies have already investigated the benefits of HIIT in exercise capacity [33].

VO_2peak_ is the gold standard method to assess the aerobic exercise capacity [31, 34]. In our meta-analysis of patients with CAD, HIIT showed a superiority compared with MICT in improving the VO_2peak_ of patients. Given the significant heterogeneity found in the primary analyses due to the variance in exercise protocols (variable intensities and different durations of the exercise programs), caution is warranted when interpreting our results. Our finding showed that HIIT resulted in a larger gain of 1.50 mL/kg/min on VO_2peak_ than MICT, and these results are in line with previous meta-analyses [8–10].

According to the duration of the total intervention, our research showed that <12 weeks group resulted in a greater improvement in VO_2peak_ by 2.75 mL/kg/min in MD than ≥ 12 weeks group did, which is in line with Taylor et al.'s finding [35], which reported home-based HIIT and MICT had low rates of adherence features compared with the supervised stage. Only one included trial [21] stated the protocol consisted of 6 supervised sessions (4 weeks) and 24 unsupervised sessions for an additional 8 weeks (12 weeks total). Therefore, higher patient acceptance of short-term exercise may have contributed to this outcome.

A meta-analysis involved one million adults suggested that 10 mmHg decrease of SBP and DBP could reduce the risk of premature death from stroke and ischemic heart disease by 40% and 30%, respectively [36]. In patients with hypertension, both HIIT and MICT reduced ambulatory blood pressure, increasing the percentage of patients with normal ambulatory blood pressure values [37]. However, no significant changes were found in our meta-analysis of SBP and DBP after HIIT and MICT intervention. With the reason for the significant heterogeneity among studies being unknown, whether there was a significantly greater effect on blood pressure in HIIT compared with MICT is still uncertain. This may be attributed to the inclusion of CAD patients rather than hypertensive patients in this meta-analysis. It seems that HIIT reduced SBP better than MICT in our report. Our results are inconsistent with Du et al.'s [38], who reported MICT seemed to induce a larger reduction in both SBP and DBP than HIIT. Three [20, 23, 25] included trials reported changes in medications during the invention. This would make it difficult to interpret and discuss the underlying mechanisms. Factors associated with medications should be considered when making personalized prescriptions.

In resent epidemiological studies, Aboyans and Criqui [39] indicated that elevated HRrest is independently associated with atherosclerosis and increased cardiovascular morbidity and mortality in cardiovascular diseases. Our results suggested that HRpeak and HRrest are equally influenced by HIIT and MICT. It is suggested that vigorous exercise could increase the risk of sudden cardiac events in susceptible individuals [40]. According to the results of Rognmo et al.'s study [41], the risk of cardiovascular events is low after performing high-intensity exercise or moderate-intensity exercise in cardiovascular rehabilitation.

## 5. Strengths and Limitations

The strength of this systematic review provided an updated analysis of data from RCTs that compared HIIT to MICT in patients with CAD. Moreover, this study was conducted in compliance with the PRISMA checklist for clear reporting, registration on INPLASY platform with protocol, and applying the GRADE tool to assess the certainty of the evidence. The study has potential limitations. First, few trials reported in detail on randomization procedures to determine whether selection bias might have affected study outcomes. Another important limitation is the small number of studies comparing HIIT and MICT with isocaloric protocols. On the other hand, the pooled studies lack large-scale clinical RCTs, which may affect the objectivity and reliability of this meta-analysis. In addition, the duration of the training program ranged from 4 to 24 weeks. The long-term safety and effects of HIIT are still unknown.

## 6. Conclusion

This meta-analysis and systematic review reported the superiority of HIIT in improving VO_2peak_ in CAD patients compared with MICT. These findings suggest that HIIT is a promising alternative exercise protocol for improving cardiorespiratory function in patients with CAD. The duration of the intervention and the availability of supervision are further considerations for the exercise protocols. Moreover, there was no difference between the HIIT and MICT effects on SBP and DBP or peak and resting HR. In further studies, larger and longer-term studies are needed to address inadequate evidence.

## Figures and Tables

**Table 1:** Characteristics of included studies.

Study	Sample size	Ages	Training protocols		Program duration
	*T*/*C* (M/F)	*T*/*C*	HIIT	MICT	
Cardozo et al., 2015	23 (14, 9)/48 (34, 14)	*T*: 56 ± 12	10 bouts ∗ 2 min (>90% HRpeak)	30 min of continuous training (at 70 to 75% of HRpeak)	16 weeks
		*C*: 62 ± 12	Each interval: 2 min (<60% HRpeak)		

Choi et al., 2018	23 (21, 2)/21 (18,)	*T*: 60 ± 11	4 bouts ∗ 4 min (at 85–100% of the HRpeak)	28 min of continuous training (at 60 to 70% of HRpeak)	9-10 weeks
		*C*: 62.8 ± 11.9	Each interval: 3 min (at 50–60% of the HRpeak)		

Conraads et al., 2015	85 (NA)/89 (NA)	NA	4 bouts ∗ 4 min (at 85–90% of peak VO2	37 min of continuous training (at least 60–70% of peak VO2, at least 65–75% of HRpeak)	12 weeks
			90–95% of HRpeak, 15–17 Borg scale, and shortness of breath)		
			Each interval: 3 min (at 50–70% of HRpeak)		

Currie et al.,2013	11 (NA)/11 (NA)	*T*: 63 ± 8	Part 1 (week 1–4): 10 bouts ∗ 1 min (at 89% of PPO pre)	Part1 (week 1–4): 30 min of continuous training (at 58% of PPOpre)	12 weeks
		*C*: 66 ± 8	Part 2 (week 5–8): 10 bouts ∗ 1 min (at 102% of PPOpre)		
			Part 3 (week 9–12): 10 bouts ∗ 1 min (at 110% of PPOpre)	Part 2 (week 5–8): 40 min of continuous training (at 58% of PPOpre)	
			Each interval: 1 min (at 10% of PPOpre)	Part 3 (week 9–12): 50 min of continuous training (at 58% of PPOpre)	

Currie et al., 2014	9 (9)/10 (9, 1)	*T*: 62 ± 11 *C*: 68 ± 8	Part 1 (month 1): 10 bouts ∗ 1 min (at 85% of PPOpre)	Part 1 (month 1): 30 min of continuous training (at 57% PPOpre)	24 weeks
			Part 2 (month 2): 10 bouts ∗ 1 min (at 100% of PPOpre)		
			Part 3 (month 3): 10 bouts ∗ 1 min (at 108% of PPOpre)	Part 2 (month 2): 40 min of continuous training (at 57% PPOpre)	
			Part 4 (month 4–6): 10 bouts ∗ 1 min (at 121% of PPOpre)	Part 3 (month–3): 50 min of moderate-intensity exercise (at 57% PPOpre)	
			Each interval: 1 min (at 10% of PPOpre)	Part 4 (month4–6): min of continuous training (at 78% PPOpre)	

Dunford et al., 2021	9/11 (Total: 18/2)	Total: 61 ± 7	3 bouts ∗ 90 s stairs climbing	30 min of continuous training (at 60–80% HRpeak)	12 weeks
			Each interval: walking 90 s		

Jaureguizar et al., 2016	36 (33, 3)/36 (28, 8)	*T*: 58 ± 11 *C*: 58 ± 11	Part 1 (week 1): HIIT: 15 bouts ∗ 20 s (50% of the maximum load reached in the first SRT)	Part 1: MICT: 15 mins (at (VT1)	8 weeks
			Each interval: 40 s (10% of the maximum load reached in the first SRT)	Part 2 (2 w): MICT: 20 mins (at VT1)	
			Part 2 (week 2): HIIT: 20 bouts ∗ 20 s (50% of the maximum load reached in the first SRT)	Part 3 (3 w): MICT: 25 mins at (VT1)	
			Each interval: 40 s (10% of the maximum load reached in the first SRT)	Part4 (4 w): MICT: 30 mins at (VT1)	
			Part 3 (week 3): HIIT: 25 bouts ∗ 20 s (50% of the maximum load reached in the first SRT)	Part 5 (5–8 w): MICT: 30 mins at (VT1+10%)	
			Each interval: 40 s (10% of the maximum load reached in the first SRT)		
			Part 4 (week 4): HIIT: 30 bouts ∗ 20 s (50% of the maximum load reached in the first SRT)		
			Each interval: 40 s (10% of the maximum load reached in the first SRT)		
			Part5 (week 5–8): HIIT: 30 bouts ∗ 20 s (50% of the maximum load reached in the second SRT)		

Keteyian et al., 2014	36 (33, 3)/36 (28, 8)	*T*: 58 ± 11 *C*: 58 ± 11	4 bouts of 4 min (at 80–90% of the heart rate reserve)	30 min of continuous training (at 60% to 80% of heart rate reserve)	2 weeks
			Each interval: 3 min (at 60–70% of the heart rate reserve)		

Kim 2020	23 (18, 5)/24 (16, 8)	*T*: 60 ± 11	4 bouts ∗ 4 min (at 95–100% of the HRR)	First part: 3 bouts ∗ 8 min (at 85% of the HRR)	6 weeks
		*C*: 62.8 ± 11.9	Each interval: 3 min (at 60% of the HRR)	Each interval: 3 min (at 40% of the HRR)	

Moholdt et al., 2009	28 (24, 4)/31 (24, 7)	*T*: 60.2 ± 6.9	4 bouts ∗ 4 min (at 90% of the HRpeak)	46 min of continuous training (at least 70% of HRpeak)	4 weeks
		*C*: 62.0 ± 7.6	Each interval: 3 min (70% of the HRpeak)		

Moholdt et al., 2012	30 (25, 5)/59 (49, 10)	*T*: 56.7 ± 10.4	4 bouts ∗ 4 min (at 85–95% HRpeak)	Usual care exercise: 60 min of aerobic exercises	12 weeks
		*C*: 57.7 ± 9.3	Each interval: 1 min (70% HRpeak)		

Pattyn et al., 2016	80 (76, 4)/83 (76, 7)	*T*: 57.4 ± 8.7	4 bouts of 4 min (at 85–95% of the HRpeak)	37 min of continuous training (at least 70–75% of HRpeak)	12 weeks
		*C*: 59.9 ± 9.2	Each interval: 3 min (50%–70% of the HRpeak)		

Prado et al., 2016	17(14, 3)/18 (14, 4)	*T*: 56.5 ± 2.7	7 bouts ∗ 3 min (at RCP)	50 min of continuous training (at VAT)	12 weeks
		*C*: 61.3 ± 2.2	Each interval: 3 min (at VAT)		

Rocco et al., 2012	17(14, 3)/20 (15, 5)	*T*: 56.5 ± 3.0	7 bouts ∗ 3 min (at RCP)	50 min of continuous training (at VAT)	12 weeks
		*C*: 62.3 ± 2.0)	Each interval: 3 min (at VAT)		

Rognmo et al., 2004	8(6, 2)/9(8, 1)	*T*: 62.9 ± 11.2	4 bouts ∗ 4 min (at 80–90% oVO2peak (85–95% of HRpeak)	41 min of continuous training (at 50–60% of VO2 peak)	10 weeks
		*C*: 61.2 ± 7.3	Each interval: 3 min (at 50–60% of VO2peak)		

Warburton et al., 2005	7(NA)/7(NA)	*T*: 55.9 ± 7	15 bouts ∗ 2 min (at 90% of heart rate/VO2reserve (range 85% to 95%))	30 min of continuous training (at 65% of heart rate/VO2reserve)	16 weeks
		*C*: 57 ± 8	Each interval: 2 min (at 40% of heart rate/VO2reserve (range 35% to 45%))		

## Data Availability

The data used to support the findings of this study are available from the authors upon request.

## Abbreviations

CAD: Coronary artery disease
CR: Cardiac rehabilitation
HIIT: High-intensity interval training
MICT: Moderate-intensity continuous exercise
VO_2peak_: Peak oxygen consumption
HRpeak: Heart rate
HRrest: Resting heart rate
SBP: Systolic blood pressure
DBP: Diastolic blood pressure
RCTs: Randomized controlled trials.

## Supplementary Materials

Supplementary Materials (1) Table S1: search strategy in English databases. (2) Figure S1: funnel plot of publication bias. (3) PRISMA 2009 Checklist.
